# Extensive mucosal sloughing of the small intestine and colon in a patient with severe COVID‐19

**DOI:** 10.1002/deo2.42

**Published:** 2021-09-07

**Authors:** Tsukasa Yamakawa, Keisuke Ishigami, Ayumu Takizawa, Yumemi Takada, Sae Ohwada, Yoshihiro Yokoyama, Tomoe Kazama, Daisuke Hirayama, Shinji Yoshii, Hiro‐O Yamano, Rina Ohizumi, Naofumi Bunya, Taro Sugawara, Mitsuhiro Tsujiwaki, Shintaro Sugita, Satoshi Takahashi, Eichi Narimatsu, Hiroshi Nakase

**Affiliations:** ^1^ Department of Gastroenterology and Hepatology Sapporo Medical University Hokkaido Japan; ^2^ Department of Emergency Medicine Sapporo Medical University Hokkaido Japan; ^3^ Department of Surgical Pathology Sapporo Medical University Hokkaido Japan; ^4^ Department of Infection Control and Laboratory Medicine Sapporo Medical University Hokkaido Japan

**Keywords:** capsule endoscopy, COVID‐19, mucosal sloughing

## Abstract

Patients with coronavirus disease 2019 (COVID‐19) primarily cause respiratory symptoms. However, gastrointestinal (GI) symptoms can also occur. The endoscopic characteristics of the GI tract in COVID‐19 patients remain unclear. We herein report a 62‐year‐old male with severe COVID‐19 who needed multidisciplinary treatment, including extracorporeal membrane oxygenation (ECMO). Despite the improvement in his respiratory status, GI bleeding developed. Capsule endoscopy and colonoscopy revealed extensive mucosal sloughing in the lower intestinal tract. Additionally, we performed a comprehensive analysis of the mRNA expression levels of various proinflammatory cytokines in the intestinal mucosal tissues. The results suggested a significant elevation of *IL‐6*, which could be involved in the pathophysiology of the GI involvement in COVID‐19. Further investigation with more clinical data, including endoscopic findings and molecular analyses, will contribute to a comprehensive understanding of COVID‐19‐associated GI injury.

## INTRODUCTION

Patients with coronavirus disease 2019 (COVID‐19) primarily causes respiratory symptoms. However, gastrointestinal (GI) symptoms can also occur. Although several reports have indicated an association between the severity of COVID‐19 and the frequency of GI symptoms, such as diarrhea and abdominal pain,^1^ the endoscopic characteristics of the GI tract in COVID‐19 patients remain unclear. The clinical features in COVID‐19 patients with GI involvement could help us understand the pathophysiology of SARS‐CoV‐2 infection in the GI tract. We herein report a patient with severe COVID‐19 who had extensive mucosal sloughing in the lower intestinal tract detected by capsule endoscopy and colonoscopy, and mucosal cytokine analysis suggested a significant elevation of *IL‐6*, which could be involved in the pathophysiology of the GI involvement in COVID‐19.

## CASE REPORT

**FIGURE 1:**
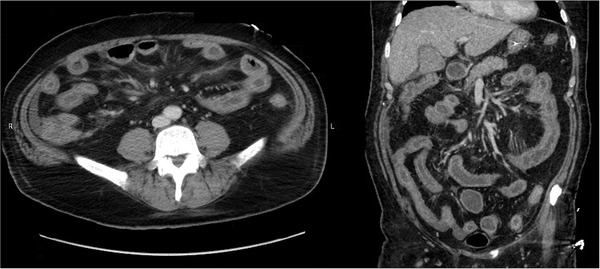
Contrast‐enhanced CT showed mucosal contrast effect and submucosal edema in the entire small intestine and the colon. And there was no decreased contrast effect of the gut suspect of ischemic change or thrombi in the abdominal vessels

A 62‐year‐old male with a history of hypertension and diabetes mellitus was transferred from another hospital to the emergency department of our hospital with a 2‐day history of fever, chills, and fatigue. A nasopharyngeal swab tested positive for SARS‐CoV‐2. A chest CT scan showed diffuse ground‐glass opacities in the bilateral lobes. Despite the administration of favipiravir (AVIGAN) and tocilizumab (ACTEMRA), his respiratory condition gradually deteriorated over the course of 2 weeks; therefore, he was transferred to our hospital for treatment with extracorporeal membrane oxygenation (ECMO). Although his pneumonia gradually improved on ECMO, he had a significant amount of watery stool with bleeding. His abdomen was soft, with no tenderness. Routine blood tests showed a white blood cell count of 4900/μl, hemoglobin level of 12.4 g/dl, platelet count of 45,000/μl, C‐reactive protein level of 3.59 mg/dl, fibrinogen level of 129 mg/dl (200‐400 mg/dl), and D‐dimer level of 26.2 μg/ml (< 1.0 μg/ml). The stool culture and tests for pathogens were all negative. Contrast‐enhanced CT showed submucosal edema in both the small intestine and colon without any decreased contrast effect of the gut suspect of ischemic change or thrombi in the abdominal vessels (Figure 1). We performed an endoscopic examination to confirm the GI findings on CT.

During endoscopy, all endoscopists and assistants thoroughly implemented standard precautions, using caps, face shields, N95 masks, gloves, and gowns. Tissue samples collected from the COVID‐19 patient were transported by one doctor in triple packaging, according to the guidelines of the National Institute of Infectious Diseases. We performed the subsequent laboratory procedures in a Biosafety level 2.

**FIGURE 2:**
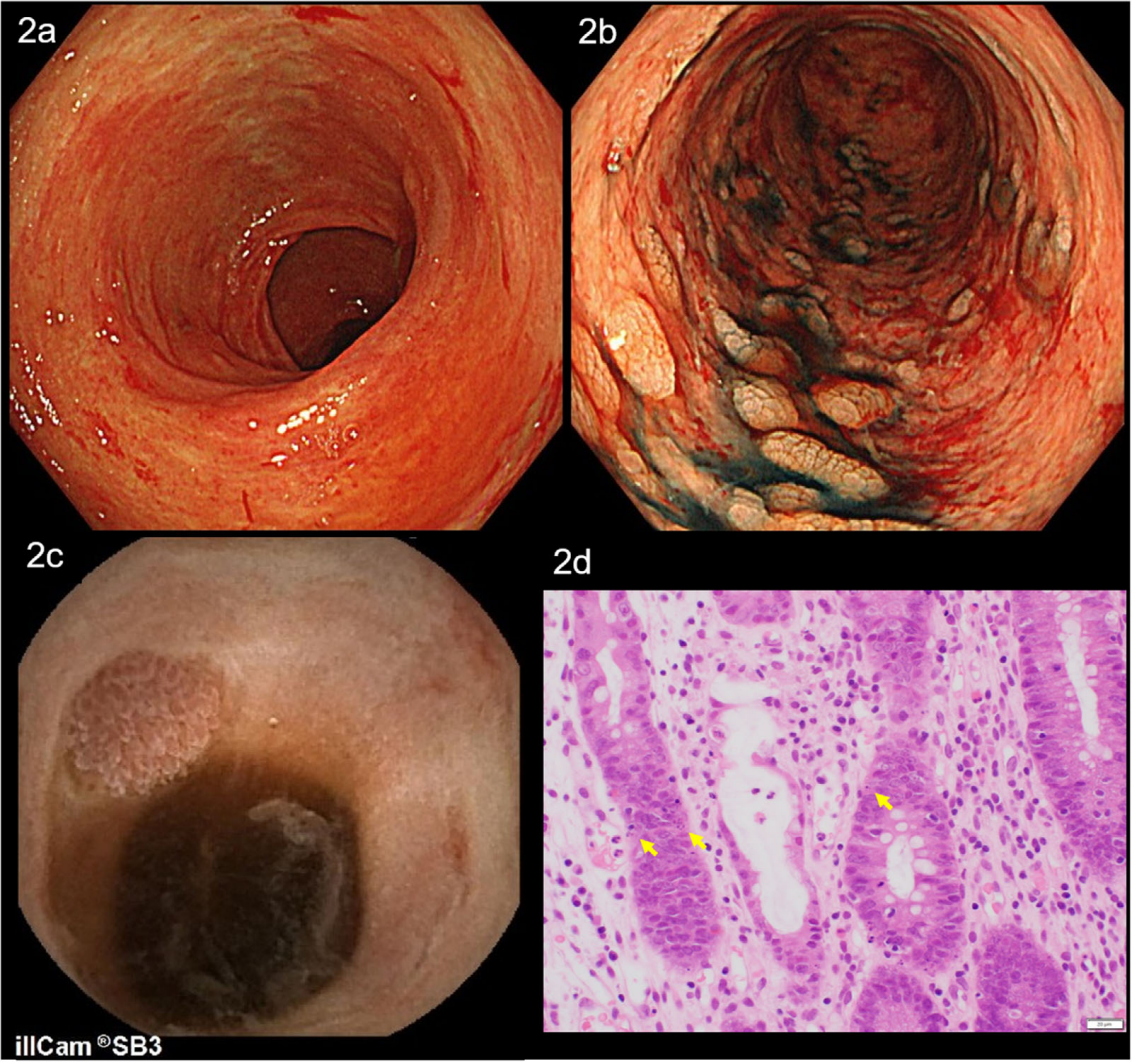
Colonoscopy with indigo carmine dye spraying showed extensive mucosal sloughing in the (a) terminal ileum and the (b) descending colon. Capsule endoscopy showed extensive mucosal sloughing in the entire (c) small intestine. The biopsy specimen from the colonic tissue showed mucosal erosion, edema, infiltration of lymphocyte dominant inflammatory cells, and apoptosis of the enterocytes (d, arrows: apoptotic bodies; HE × 400)

Gastroduodenoscopy and colonoscopy showed extensive mucosal sloughing in the 3rd to 4th portion of the duodenum and from the terminal ileum (Figure 2a) to the descending colon (Figure 2b). There were multiple ulcers in the sigmoid colon and rectum. Next, we performed capsule endoscopy (PillCam SB3 capsule, COVIDIEN, Dublin, Ireland). It showed extensive mucosal sloughing throughout the entire small intestine (Figure 2c), which mimicked severe graft‐versus‐host disease (GVHD). The biopsy specimens from the colonic tissue showed mucosal erosion, edema, infiltration of lymphocyte dominant inflammatory cells, and apoptosis of the enterocytes (Figure 2d). There were no microthrombus, any signs of specific bacterial infection, and viral inclusion bodies, suggestive of cytomegalovirus infection.

**FIGURE 3:**
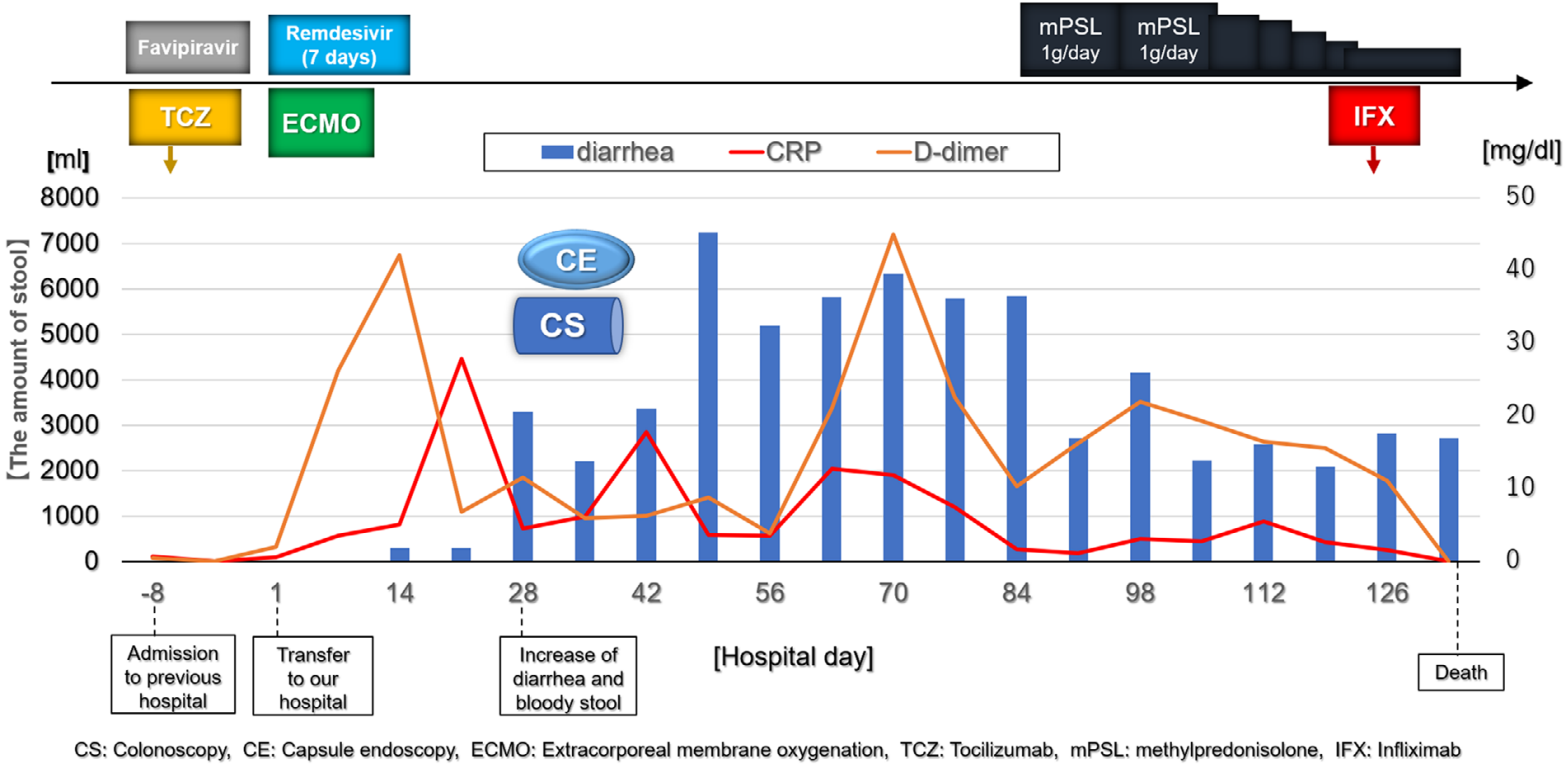
Clinical time course from the onset of COVID‐19 pneumonia to the death, including the day of the colonoscopy, capsule endoscopy, the medications, and the transition of laboratory data (CRP, D‐dimer) and the amount of stool

Although steroid pulse therapy with 1 g/day methylprednisolone (mPSL) for 3 days led to temporary improvements in his diarrhea and hematochezia, these GI symptoms returned to previous levels after the cessation of treatment. Next, we administered 5 mg/kg infliximab (Remicade) twice with an intervening interval of 2 weeks; however, he did not respond to the treatment. Finally, he died of multiple organ failure (Figure 3).

## DISCUSSION

This patient with severe COVID‐19 needed multidisciplinary treatment, including ECMO. Despite the improvement in his respiratory status, GI bleeding developed. Endoscopic examination revealed mucosal sloughing of both the entire small intestine and colon, mimicking severe GVHD. Unfortunately, mPSL and anti‐TNFα antibody administration did not improve his condition. To our knowledge, this is the first endoscopic description of extensive mucosal injury in the small and large bowel in a patient with severe COVID‐19. It is possible to gain an understanding of the pathophysiology of GI mucosal injury related to SARS‐CoV‐2 infection through this case.

Reports of non‐respiratory manifestations, particularly GI symptoms in COVID‐19 patients, have increased.^1^ The typical symptoms of patients with GI involvement of COVID‐19 include diarrhea and nausea, while GI bleeding is rare. A pooled analysis of multiple studies evaluating the correlation between GI symptoms and COVID‐19 severity demonstrated that abdominal pain was associated with four‐fold higher odds of severe disease.^1^ However, the association between COVID‐19 severity and GI bleeding has not been addressed. Moreover, there have been few reports of the characteristics about endoscopic findings in COVID‐19 patients with GI involvement.

The putative mechanisms underlying the development of GI symptoms in COVID‐19 patients are as follows: (1) A viral cytopathic effect, (2) viral‐induced inflammation, (3) hypo‐perfusion due to microthrombosis or life‐threatening condition itself, (4) altered gut microbiota, and (5) secondary infections. Among these mechanisms, there is a possibility that the severe mucosal damage in both the small intestine and colon, in this case, was due to the coexistence of hypo‐perfusion and viral‐induced inflammation.

Generally, SARS‐CoV‐2 enters enterocyte, and then viral synthesis and replication occur, which leads to a cytopathic effect (evidenced by the intracellular staining of viral nucleocapsids).^2^ Additionally, virus‐derived nucleic acids activate pattern recognition receptors in virally infected cells, and innate immunity is subsequently activated. Similar to the SARS‐CoV‐1 and MERS‐CoV infection mechanisms, inflammatory cytokines such as TNF‐α, IL‐1, and IL‐6 are activated.^3^ Subsequently, several immune cells such as macrophages, neutrophils, T cells, and innate lymphoid cells are stimulated by these cytokines to produce proinflammatory cytokines. Thus, SARS‐CoV‐2 can exert its cytopathic/inflammatory effects after entry into cells in the GI tract.

**FIGURE 4:**
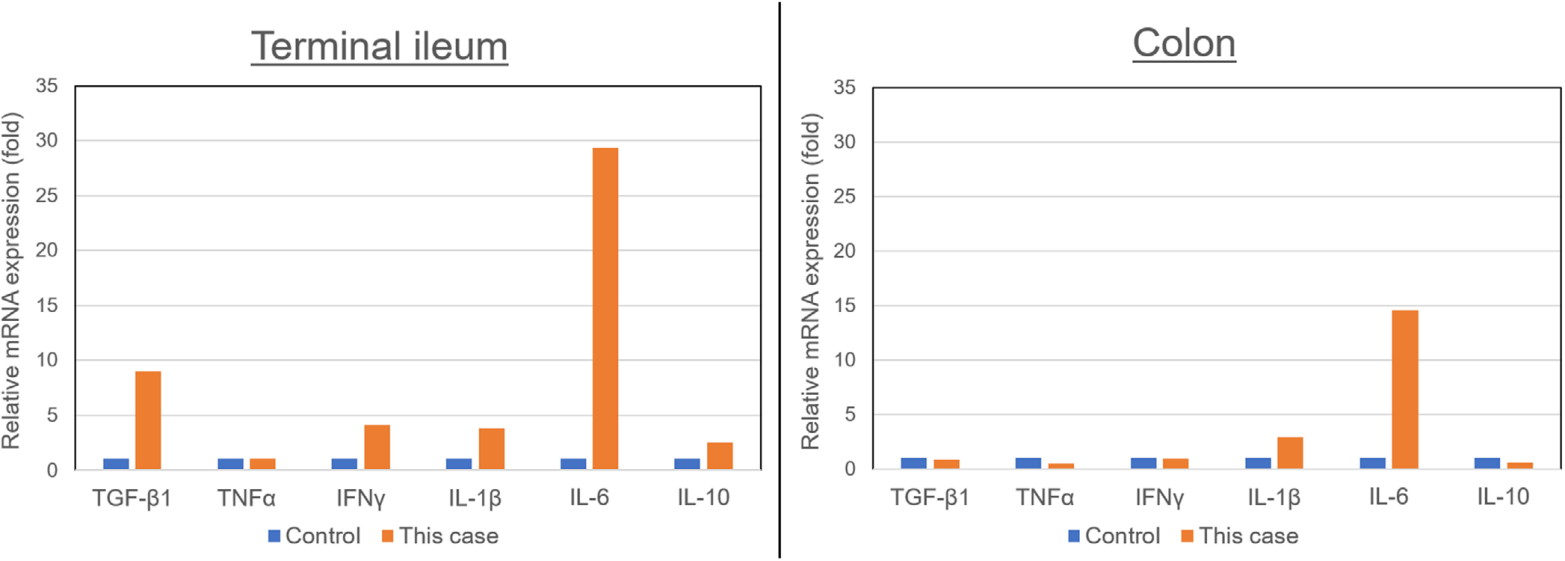
Comprehensive analysis of inflammatory cytokines using the RT2 Profiler PCR Array showed that the expression level of *IL‐6* was obviously elevated in both the terminal ileum and the colon compared to in the control samples (we used a colonic mucosa in a patient with ulcerative colitis in remission as control sample)

Additionally, we comprehensively analyzed the mRNA expression levels of various proinflammatory cytokines in this patient's intestinal mucosal tissue using the RT2 Profiler PCR Array (Human Inflammatory Cytokines and Receptors manufactured by QIAGEN, Venlo, Netherlands). We used a colonic mucosa in patients with ulcerative colitis (UC) in remission as control. The results showed increased expression levels of *IL‐6* in both the small intestine and the colon, which suggested an IL‐6 dominant cytokine reaction in the GI tract (Figure 4).

IL‐6 is a proinflammatory cytokine produced by macrophages, T cells, B cells, fibroblasts, and vascular endothelial cells. In patients with severe COVID‐19 requiring intensive care, higher blood plasma levels of several proinflammatory cytokines have been observed.^4^ Among them, IL‐6 is considered the key driver of the hyperinflammatory process in COVID‐19, as supported by a meta‐analysis of six studies demonstrating 2.9‐fold higher mean IL‐6 concentrations in patients with complications of COVID‐19 than in those without complications.^5^ Therefore, selective immunosuppression with drugs such as anti‐IL‐6 antibody is thought to be beneficial with regard to pulmonary hyperinflammation in patients with COVID‐19, also called “cytokine release syndrome”.^6^ In this case, the patient received tocilizumab, which may have improved his systemic inflammation.

On the other hand, the patient was negative for SARS‐CoV‐2 PCR test using stool sample. Additionally, a previous report demonstrated that IL‐6 is essential for the proliferation and regeneration of epithelial cells.^7^ Therefore, we considered that the increase in the level of IL‐6 in small intestinal and colonic tissues might reflect epithelial regeneration rather than sustained SARS‐CoV‐2 infection. Also, there is a report that an inverse association between pre‐treatment *TNFα* expression in colorectal mucosa and both clinical and endoscopic response of infliximab in UC patients.^8^ Taken together, we administered infliximab for this patient to treat this mucosal sloughing.

As experience with COVID‐19 has expanded, it has been reported that patients with COVID‐19 have thrombotic microvascular injury of a systemic nature and that the lung, skin, kidney, GI tract, and other organs are affected.^9^ However, it remains unclear whether COVID‐19‐related coagulopathy is a by‐product of a severe inflammatory response or a virus‐specific type of coagulopathy. Buckholz et al. reported COVID‐19‐related microthrombotic disease of the small intestine based on endoscopic findings such as edema, granularity, and patchy erythema.^10^ In the present case, COVID‐19‐related microthrombosis was the possible mechanism underlying the severe mucosal damage, despite the fact that no thrombus was detected.

In conclusion, we present the case of a COVID‐19 patient with severe mucosal damage in both the small intestine and colon. The pathophysiology of COVID‐19‐associated GI mucosal injury has not yet been clarified. Further investigation with more clinical data, including endoscopic findings and molecular analyses, will contribute to a comprehensive understanding of COVID‐19‐associated GI injury.

## CONFLICT OF INTERESTS

No authors have any conflict of interest to declare.

## FUNDING INFORMATION

None.

## ETHICAL STATEMENT

Written informed consent based on the Helsinki Declaration (1964, 1975, amended in 1983, 2003, and 2008) was obtained from all patients. The Review Board of Sapporo Medical University reviewed and approved the study protocol.
